# Development of an Internet-Administered Cognitive Behavior Therapy Program (ENGAGE) for Parents of Children Previously Treated for Cancer: Participatory Action Research Approach

**DOI:** 10.2196/jmir.9457

**Published:** 2018-04-18

**Authors:** Anna Wikman, Laura Kukkola, Helene Börjesson, Martin Cernvall, Joanne Woodford, Helena Grönqvist, Louise von Essen

**Affiliations:** ^1^ Clinical Psychology in Healthcare Department of Women's and Children's Health Uppsala University Uppsala Sweden

**Keywords:** cognitive therapy, psychology, clinical, e-therapy, community participation, Sweden

## Abstract

**Background:**

Parenting a child through cancer is a distressing experience, and a subgroup of parents report negative long-term psychological consequences years after treatment completion. However, there is a lack of evidence-based psychological interventions for parents who experience distress in relation to a child’s cancer disease after end of treatment.

**Objective:**

One aim of this study was to develop an internet-administered, cognitive behavior therapy–based, psychological, guided, self-help intervention (ENGAGE) for parents of children previously treated for cancer. Another aim was to identify acceptable procedures for future feasibility and efficacy studies testing and evaluating the intervention.

**Methods:**

Participatory action research methodology was used. The study included face-to-face workshops and related Web-based exercises. A total of 6 parents (4 mothers, 2 fathers) of children previously treated for cancer were involved as parent research partners. Moreover, 2 clinical psychologists were involved as expert research partners. Research partners and research group members worked collaboratively throughout the study. Data were analyzed iteratively using written summaries of the workshops and Web-based exercises parallel to data collection.

**Results:**

A 10-week, internet-administered, cognitive behavior therapy–based, psychological, guided, self-help intervention (ENGAGE) was developed in collaboration with parent research partners and expert research partners. The content of the intervention, mode and frequency of e-therapist support, and the individualized approach for feedback were modified based on the research partner input. Shared solutions were reached regarding the type and timing of support from an e-therapist (eg, initial video or telephone call, multiple methods of e-therapist contact), duration and timing of intervention (eg, 10 weeks, 30-min assessments), and the removal of unnecessary support functions (eg, removal of chat and forum functions). Preferences for study procedures in future studies testing and evaluating the intervention were discussed; consensus was not reached for all aspects.

**Conclusions:**

To the best of our knowledge, this study is the first use of a participatory action research approach to develop a psychological intervention for parents of children previously treated for cancer and to identify acceptable study procedures. Involvement of parents with lived experience was vital in the development of a potentially relevant and acceptable intervention for this population.

## Introduction

### Background

Although majority of the children diagnosed with cancer survive their disease [1], childhood cancer impacts the entire family from cancer diagnosis to survivorship [2]. For parents, a child’s treatment completion represents an important milestone, but it also represents a period of psychological vulnerability [3,4]. Indeed, a subgroup of parents report negative long-term psychological consequences years after completion of treatment [4-6]. However, currently, there is a lack of evidence-based psychological interventions for parents who experience distress in relation to a child’s cancer disease after end of treatment. Recently published clinical guidelines, outlining how children diagnosed with cancer and their family members should be cared for, recommend referrals to appropriate psychosocial and therapeutic support into long-term survivorship [7]. Despite these recommendations, we have recently shown that subgroups of parents report an unmet need of psychological support after end of treatment [8]. Furthermore, although face-to-face cognitive behavior therapy (CBT) shows promise in decreasing post-traumatic stress symptoms (PTSS), depression, and anxiety among parents of children previously treated for cancer (personal communication by L Ljungman, 2017-10-23), challenges remain regarding provision of psychological support to those parents of children previously treated for cancer who need such support. Indeed, our recent findings showing an unmet need of psychological support among parents of children previously treated for cancer [8] are in line with findings from one study in Australia showing that formal psychological support was difficult to access and rarely received by parents after cancer treatment completion [9]. This study concluded that factors related to staff availability, models of assessment and delivery of services, and size and location of pediatric cancer centers may hinder the provision of support.

Provision of CBT via the internet may increase access to psychological support and may be an alternative for parents of children previously treated for cancer. One previous study has shown high acceptability and feasibility of a Web-based, group-based, CBT intervention, delivered “live” by a psychologist, for parents following cancer treatment completion [10]. In a randomized controlled trial (RCT), we have shown a Web-based psychological self-help intervention to be effective in reducing PTSS, depression, and anxiety among parents of children recently diagnosed with cancer [11], with improvements maintained at 1-year follow-up [12]. This RCT was initiated in 2010, and the intervention, although Web-based, utilized few technological features, as it provided material in the form of PDF files and secure written communication between the participant and therapist. During this trial, challenges, such as low enrolment rate and considerable attrition, were identified [11,12]. Factors to enhance inclusion and retention rates such as end-user involvement when developing interventions and study procedures to test and evaluate interventions for parents of children with cancer have been suggested as essential next steps for intervention research [13]. Furthermore, others have put forward that internet-administered, CBT-based, self-help interventions should be developed with the target population in mind [14]. Indeed, poorer levels of acceptability have been found for internet-administered interventions not developed for and tailored toward specific populations [15]. Additionally, research suggests recruitment and adherence rates may be improved if the perspective of the population is adopted [16].

### Objective

Due to previous findings [4-6,8] as well as challenges with recruitment and attrition when offering parents psychological support during the child’s treatment, it was decided to focus subsequent research on parents later during their child’s disease trajectory, specifically after the end of treatment. Building on our group’s previous work with this population (personal communication by L Ljungman, 2017-10-23) [4,5], representing the Medical Research Council (United Kingdom) phase I (development) research [17], this study adopted a participatory action research (PAR) approach [18]. One aim of this study was to develop an internet-administered, CBT-based, psychological, guided, self-help intervention (ENGAGE) for parents of children previously treated for cancer. Another aim was to identify acceptable procedures for future feasibility and efficacy studies testing and evaluating the intervention.

## Methods

### Design and Setting

The study was carried out according to PAR, which is a collaborative process of knowledge production and colearning, placing people with lived experience at the center of the process [18]. A group of people with lived experience of parenting a child previously treated for cancer were involved as parent research partners (PRPs) and took part in 8 workshops and related Web-based exercises. The study was carried out at Uppsala University, Uppsala, Sweden, and was conducted over an 8-month period during 2016 on weekday evenings based on PRPs’ preferences. An additional workshop was carried out during this period with 2 expert research partners (ERPs), both clinical psychologists and experts in internet-administered psychological interventions. Ethical approval was granted by the regional ethical review board in Uppsala, Sweden (Dnr: 2015/426).

### Research Partners

#### Parent Research Partners

Eligible PRPs were the ones who (1) lived near Uppsala (≤100 km), (2) spoke Swedish, (3) were a parent of a child previously treated for cancer, and (4) had experienced or were experiencing psychological distress related to the child’s cancer disease. Current severe psychological distress (eg, symptoms of a severe and enduring mental health difficulty, misuse of alcohol or drugs, acutely suicidal) excluded parents from participation. Parents of children previously treated for cancer who had participated in our group’s previous intervention research [11,12] (personal communication by L Ljungman, 2017-10-23) were invited strategically via letter and a telephone call, considering variation in gender and socioeconomic status. If 2 parents of 1 child had participated in a previous study, then only 1 parent was invited. This was to avoid overrepresentation of 1 family’s experience in the group, and to achieve an open discussion environment, which may have been influenced by the presence of one’s partner. In addition, information about the study was posted on the Swedish Childhood Cancer Foundation Website and the Swedish Childhood Cancer Association Middle-Sweden Facebook page to increase awareness of the study, including an open invitation to participate in the study. The sample consisted of 6 parents, 5 of whom had participated in our previous intervention research. PRPs participated outside their regular working hours and were reimbursed for time (fixed amount for workshops and Web-based assignments completed) and travel expenses at the end of the study.

#### Parent Research Partner Characteristics

All eligible parents who provided informed written consent were included as PRPs (4 mothers, 2 fathers). All PRPs reported living with a partner and the majority (n=4) reported having completed a university degree. Their mean age was 50 years (SD 3), the child’s average age when diagnosed was 9.1 years (SD 4), and mean time since end of treatment was 5 years (SD 3). On average, the PRPs completed an average of 5 workshops (of 8) and 7.8 Web-based exercises (of 8).

#### Expert Research Partners

Two clinical psychologists with expertise in development and clinical use of internet-administered CBT programs were involved as ERPs. One of the ERPs had extensive experience of working with children and their family members. The ERPs were reimbursed for time spent reviewing the materials and participating in the ERP workshop and travel expenses.

### Aspects of the Intervention and Procedures for Future Studies Set at the Start of the Study

Some aspects regarding the intervention and procedures were preset before the start of the study by the research group and communicated to the PRPs and ERPs. First, the intervention should be informed by evidence-based knowledge concerning psychological distress experienced by parents of children treated for cancer and conceptualization and treatment of this distress [4,5,11,12] (personal communication by L Ljungman, 2017-10-23). Second, the intervention should be delivered via the U-CARE portal (Portal). The Portal is a secure infrastructure developed by our research group and includes functions such as log-in via bank-issued electronic identification; provision of internet-administered, guided, self-help material; communication between participants and e-therapists via internal messages and homework reports; video calls; collection of questionnaire data at predefined observation points; logging of participant behavior; Web-based library; participant chat; forum; diary; and a question and answer (Q&A) function. Third, the procedures should be possible to carry out considering the available resources and should follow national ethical research regulations.

### Procedure

#### Workshops and Web-Based Exercises With Parent Research Partners

Table 1 shows an overview of the PAR process. The workshops with PRPs were facilitated by one parent of a child previously treated for cancer with professional experience of teaching, MSc in English (coauthor HB), and one PhD student, MSc in Clinical Psychology (coauthor LK). The facilitators were responsible for constructing the Web-based exercises and materials used, taking meeting notes during the workshops, facilitating discussions, and reviewing study materials. After each workshop, HB and LK reflected upon the process with members of the research group (coauthors MC, LvE, HG, and AW). Collaboration with PRPs included 8 workshops and related Web-based exercises. Workshops were carried out in person, and Web-based exercises were carried out individually via the Portal. Each workshop had a predefined theme to focus the discussions. 

**Table 1:** Collaboration process, workshop overview, and research partner activity carried out during May to December 2016. CBT: cognitive behavior therapy; PRP: parent research partner.

Content	Workshop 1	Workshop 2	Workshops 3 and 4	Workshops 5 and 6	Expert research partner workshop	Workshop 7	Workshop 8
Topic	Welcome	Initial engagement	Intervention	Study procedures	Treatment manual	Prototype	Evaluation
Activities	Establishing contact; Presenting the study context	Discussion of opportunities and barriers for initial engagement	Discussion of modes of delivery and support functions; Refining a CBT model	Discussion of pros and cons of randomization and acceptable study procedures	Reviewing and refining a treatment manual; Discussion of manual and draft module	Reviewing and refining a treatment prototype and materials	Summary and evaluation of participatory action research process
Web-based exercises	None	Completed by 6 PRPs	Completed by 6 PRPs	Completed by 6 PRPs	None	Completed by 5 PRPs	Completed by 6 PRPs
Present at workshop	2 workshop facilitators; 2 researchers; 4 parent research partners (PRPs)	2 workshop facilitators; 3 PRPs	Workshop 3: 2 workshop facilitators; 2 PRPs Workshop 4: 2 workshop facilitators; 4 PRPs	Workshop 5: 2 workshop facilitators; 3 PRPs Workshop 6: 2 workshop facilitators; 4 PRPs	2 expert research partners; 5 researchers	2 workshop facilitators; 5 PRPs	2 workshop facilitators; 2 researchers; 6 PRPs
Time-frame	May 2016	May 2016	May 2016	June 2016	September 2016	November 2016	December 2016

Workshops 2 to 7 and Web-based exercises were conducted in an iterative manner, including the presentation of repeated written process summaries from the previous workshop to be reviewed and discussed with PRPs at the beginning of each subsequent workshop. During the first workshop with PRPs, the primary aim was to establish contact to facilitate the group process and present the study context. In the final workshop, the overall PAR process was summarized and evaluated. Workshops 2 to 7 with PRPs were carried out according to the following 2-stage process:

First, PRPs were asked to complete individual Web-based exercises before each workshop. These generally consisted of a summary from the previous Web-based exercise and workshop (workshops 2 to 7), educational or intervention materials (videos, PowerPoint lectures, or PDF texts), and related open- and close-ended questions. At the end of each Web-based exercise, PRPs were asked to provide feedback on the materials for discussion in the subsequent workshop. PRPs could indicate if they did not wish for their feedback to be discussed at the subsequent workshop and were encouraged to suggest topics they wished to reflect on in the group. PRPs were asked to provide responses to the Web-based exercises the day before the respective workshop at the latest. On the day of each workshop, the facilitators reviewed and summarized the PRPs’ individual answers to the Web-based exercises. On the basis of this summary, areas for further investigation for each PRP workshop were decided at a research group meeting, and added to the workshop agenda.

**Figure 1:**
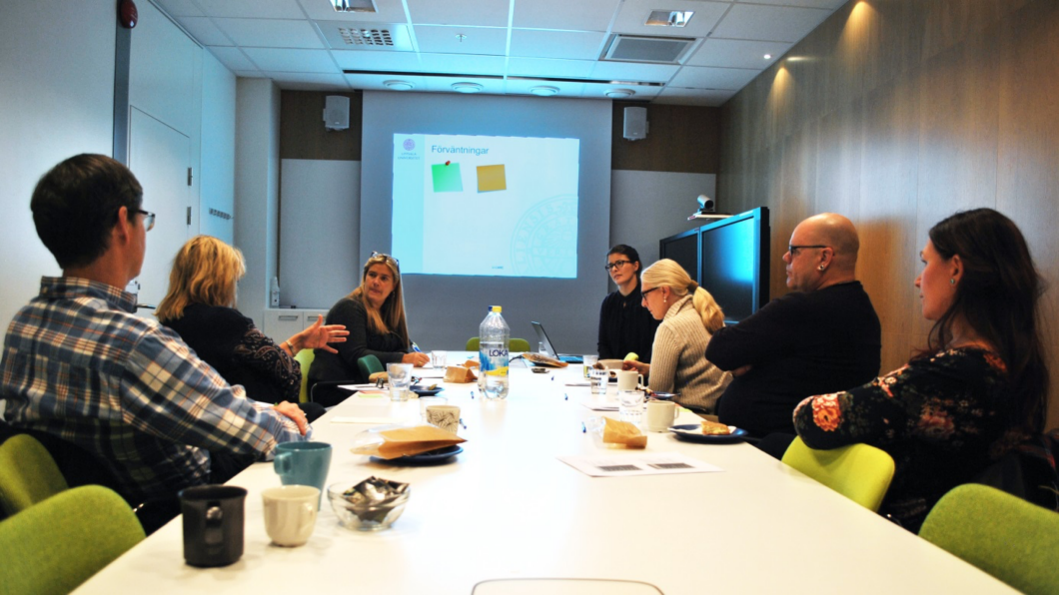
Parent research partners (PRPs) and facilitators during a workshop. Note that consent was obtained from all PRPs present to use this photo.

Second, each workshop began with a round of brief reflection, providing PRPs with the possibility to share thoughts and feelings relating to the Web-based exercise and previous workshop. Then, the agenda for the workshop was presented and PRPs were encouraged to add items to the agenda. Each workshop lasted for 2 hours, including a brief refreshment break, and consisted of individual reflection and group discussions. At the end of each meeting, an individual reflection practice was carried out where PRPs individually answered some open-ended questions. PRPs were encouraged to provide suggestions for upcoming workshops and identify the most valuable topics of the workshop. PRPs spent approximately 5 min on the task. Following this, PRPs were offered the opportunity to stay for another hour to freely reflect upon their experiences with the facilitators or to socialize with other parents, without any documentation being carried out and refreshments were provided. Figure 1 illustrates a workshop in progress.

#### Workshop With Expert Research Partners

The written intervention material (consisting of 109 A4 pages), including 1 draft module (eg, text, video, and audio materials), was presented to ERPs following the 6 workshops with PRPs. The material was written in parallel to the PRP workshops, with workshops informing the content included. During the ERP workshop, ERPs provided their expert perspectives on the material and draft module. Members of the research group (coauthors MC, LvE, HG, and AW) were present at the workshop, which was facilitated by LK. Following the workshop, the intervention material was modified, and a prototype of the intervention was presented to PRPs at workshop 7.

### Data Collection

Workshops with PRPs were documented using written meeting notes (coauthors HB and LK). Materials used for discussion practices, such as post-it notes and summaries by PRPs, were saved. At the end of each workshop (with exception of workshops 1 and 8), PRPs completed written individual process evaluations (ie, feedback on the workshops) to guide the continuation of the collaboration process. The PRPs’ responses to the Web-based exercises were documented on the Portal to ensure secure communication. During the Web-based exercises, PRPs provided feedback on extracts of the intervention and education materials presented by answering questions via the Portal. Questions focused on PRPs’ experiences of and views on aspects such as the design of the intervention materials, the Portal interface, preferences for optional support functions, and suggestions on how to improve aspects of the intervention. The ERP workshop was documented using written meeting notes from the workshop (coauthor LK, with assistance from a research assistant), with ERPs providing their written reflections on the materials after the workshop.

### Data Analysis

Data were analyzed iteratively parallel to data collection. Pragmatism was adopted as the underlying research paradigm, selecting an approach to data analysis most appropriate to the study aims [19]. Specifically, as per other studies utilizing PAR [20,21], a thematic analysis approach was adopted [22]. PRPs’ responses to the Web-based exercises were read and summarized by HB and LK and reported back to the PRPs in the subsequent workshop. Areas of further exploration in subsequent workshops were identified and discussed by the research group consisting of the research group leader (professor, PhD in clinical psychology, and clinical psychologist, coauthor LvE), 2 researchers with a PhD in psychology (coauthors HG and AW), and 2 clinical psychologists (coauthors MC and LK). Possible solutions to parents’ questions and further areas of interest were identified by the research group before each subsequent workshop. Although each workshop had a predefined theme, the agenda for each subsequent PRP workshop partially emerged from the PRPs’ Web-based exercises and reflections by the research group, with these reflections reported back to the PRPs to establish trustworthiness of the interpretations made by the research group. Furthermore, additional agenda items could be suggested by PRPs. This iterative process was used throughout the study and continued until no new themes related to study aims were identified. Over the course of the PAR process, discussions during workshops continued until data saturation was reached, that is, until no new data emerged. Following standard approaches to thematic analysis [22], data were then synthesized by members of the research group (coauthors MC, LvE and AW) firstly into descriptive and topic codes, followed by identification of themes relating to the study aims. To enhance trustworthiness, identified themes were then presented to and further discussed by the wider research group to ensure agreement on identified themes and that all data were included in the identified themes.

## Results

### Parent Research Partners’ Views on the Intervention

An overview of the results from the PAR process is shown in Table 2.

#### Duration, Content, and Presentation

PRPs stressed the importance of the intervention not being too burdensome. As such, parents suggested exercises to be shortened and provided over a longer period than originally suggested. Spending 1 hour per week working with the intervention was seen as optimal. Some suggested a duration of 7 weeks, whereas others considered a 12-week intervention better. Although consensus was not reached, a 10-week intervention was deemed acceptable.

PRPs stressed the importance of communication suggesting that the researchers should communicate clearly to potential participants in the future feasibility study that the intervention was developed to fulfill parents’ needs for psychological support, and provide clear treatment goals (eg, reducing PTSS and depression). One aspect discussed with PRPs concerned the extent to which the intervention content could be individualized, given the majority of PRPs highlighted an interest in a tailored intervention, for example, choosing specific topics to meet an individual’s particular needs. However, for the purpose of testing the feasibility and acceptability of the intervention in the upcoming feasibility study, it is important that its content remains the same for all participants.

Collaboration with PRPs resulted in including a video or telephone support call, the use of case vignettes, and replacing one of the actors in the vignettes presented to PRPs. Furthermore, the language used to describe suffering and content in modules was modified based on PRP preferences. For example, in terms of the conceptualization of distress presented during workshops 3 and 4, PRPs preferred terms such as changed life situation to depressive inactivity and difficult or painful emotions and memories rather than traumatic stress. The final version of the intervention consists of 1 introductory module followed by 10 internet-administered, guided, self-help modules.

PRPs appreciated a combination of text, audio, and video materials of high quality, including an option to print materials. The inclusion of case vignettes was valued. However, the importance of authenticity was discussed, with modifications to the vignettes made based on PRPs’ feedback on the prototype presented in workshop 7, to improve relevance and authenticity. PRPs suggested that the inclusion of an introductory video of the intervention, presented by an e-therapist and a parent, would increase trustworthiness and motivation to participate. Furthermore, an optional support function including an Web-based library containing information about CBT, self-help, literature suggestions, links to relevant Websites, as well as CBT exercises from the intervention were seen as advantageous.

**Table 2:** Overview of results from the participatory action research (PAR) process, summarizing research partners’ views on the intervention ENGAGE and acceptable procedures for future feasibility and effectiveness studies. CBT: cognitive behavior therapy.

Research partner	Views on intervention		Views on procedures	
	Theme	Description	Theme	Description
Parent research partners	Duration, content and presentation of the intervention	Not too burdensome Short Web-based exercises One hour per week 10-week program Text, audio, and video materials Language used to describe distress Case vignettes, actor changes Introductory video Web-based library including, for example, information about CBT, self-help, literature suggestions, links to relevant Websites as well as CBT exercises from the intervention	Information about study participation	Engaging and interesting information, highlighting benefits of guided self-help Information video about the study before consenting
Parent research partners	Support and contact during the interventions	Initial video or telephone session Booster video or telephone session at half-time Multiple methods of e-therapist contact (video call, telephone call, and written communication) Written feedback throughout intervention Single-item mood assessment using 5-point Likert scale to communicate changes in mood with the e-therapist	Time aspects	30 min acceptable time to complete assessments at each observation point (ie, baseline, post treatment, 6-month follow-up) 10 min acceptable time to complete weekly assessments
Expert research partners		Clear Web-based exercises Evidence-based CBT exercises Reviewing treatment goals throughout the intervention “Less is more”: intervention content reduced CBT exercises available in the Web-based library Professionally designed materials	No views provided on study procedures	

The design aspects emerging as important during the PAR process were subsequently incorporated into the design of the intervention (Table 2). Optional support functions planned for inclusion before the collaborative process included a chat function, a forum, and a Q&A function. However, these were in general viewed as unnecessary and subsequently removed from the intervention.

A written prototype of the intervention was presented to PRPs during workshop 7 and was perceived positively, with one parent stating that “parents could benefit from this.”

#### Support and Contact

At the start of the study, it was planned for the intervention to include weekly written feedback from an e-therapist trained in supporting the intervention. An e-therapist is a mental health care professional who provides support electronically, for example, via email or videoconferencing [23]. E-therapists will be psychology program students, in at least their 4th year of study, having completed a minimum of their first term of advanced studies in CBT, but will have not yet begun their prescribed practical service (ie, praktisk tjänstgöring för psykologer/PTP). PRPs highlighted the importance of personal contact via a video or telephone call with an e-therapist at the start of the intervention for parents to be able to “tell their story” and midway through the intervention to increase motivation. However, all PRPs raised the importance of parents being able to “tell their story” in their preferred way (eg, contact with the e-therapist via video or telephone call, or via written communication). As such, personal contact at the start and midway through the intervention with an e-therapist via a video or telephone call has been incorporated (Table 2). However, it was not deemed possible to completely individualize the procedure for parents “telling their story” as it is important the intervention is delivered in the same way to all participants for testing purposes in the forthcoming feasibility study.

A further issue discussed related to PRPs’ views on receiving feedback on changes in mood during the intervention. Although some considered weekly charts illustrating changes in mood as an important aspect of the intervention, the majority preferred personal feedback from their e-therapist. In the final version of the intervention, written feedback from the e-therapist is the primary method of communication as it was considered most important by PRPs. However, in line with the PRPs’ requests, a single-item mood assessment, using a 5-point Likert scale, has been incorporated as a means for participants to inform their e-therapist about their mood, in addition to the written communication.

### Expert Research Partners’ Views on the Intervention

**Figure 2:**
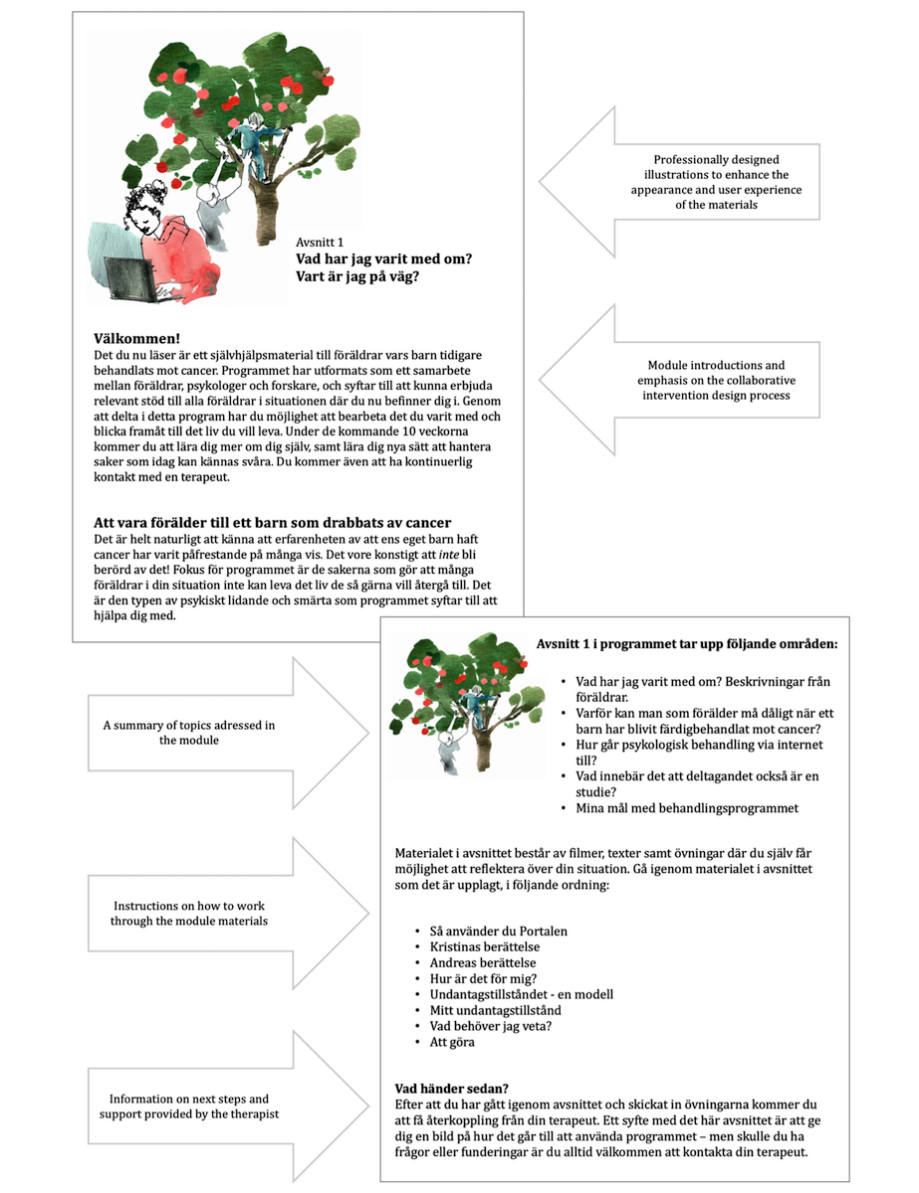
Example of an internet-administered intervention module screen (illustrations by Annika Carlsson).

Following the ERPs’ input, the intervention was further refined (Table 2). Changes included clarifying the key learning components included in each module. For example, evidence-based CBT exercises were included in each module, and weekly action plans were designed based on the content of the modules to carry out as homework. ERPs highlighted the importance of revisiting treatment goals during the intervention to maintain focus, which was subsequently more clearly incorporated in the intervention. In line with PRPs’ views, one take-home message was “less is more.” Consequently, the content in the intervention was reduced. ERPs agreed with PRPs that CBT exercises should be easily available, for example, in the Web-based library, to enable parents to return to previous exercises based on individual preferences and needs. To make the user experience more appealing, presenting a professional look, it was decided that materials and illustrations used in the intervention should be created by professionals in graphic design as suggested by the ERPs. Figure 2 shows an example of an internet-administered intervention module screen.

### Parent Research Partners’ Views on What Procedures Should be Used in Future Studies Testing and Evaluating the Intervention

#### Information About Study Participation

One key procedural aspect discussed concerned recruitment to future feasibility and effectiveness studies. PRPs stressed the importance of how study information should be provided to engage and motivate participation, highlighting the potential benefits of CBT-based, internet-administered, guided, self-help interventions. A phone call, followed by written information, was the preferred method of receiving study information. It was positively perceived to receive a study-code to access materials on the Web with further information about the study, including an informational film before consenting to participation, both of which have been included in the intervention (Table 2). PRPs were of the opinion that study information should be communicated by health care personnel, or representatives from relevant organizations, to increase trustworthiness. PRPs indicated a preference for recruitment via the hospital departments in connection with end of the child’s cancer treatment. However, as recruitment is planned for up to 5 years after end of treatment, it would not be feasible to recruit via hospital departments as parents are likely no longer in contact with the hospital clinics at that time.

#### Time Aspects

PRPs were unable to reach consensus regarding the ideal timing for the invitation to participate in a controlled study of an internet-administered, guided, self-help intervention for parents of children previously treated for cancer. Although some indicated that 3 to 6 months following end of the child’s cancer treatment would be preferable, others argued that more time must pass as parents may not be aware of their needs for psychological support so soon after end of treatment. However, all PRPs considered it important that parents are offered the participation, rather than having to seek out participation. Views differed regarding how long parents were willing to wait to receive the intervention in a hypothetical controlled study with a wait-list control group. Although a waiting period of 1 to 3 months was generally accepted, parents raised concerns about waiting when suffering emotionally following the end of their child’s treatment.

The time required to complete pre- and postintervention assessments as part of a controlled study was discussed, and PRPs considered 30 min to be an acceptable length of time for completing these assessments. In addition, spending 10 min weekly for completing the questionnaires while receiving the intervention was an acceptable amount of time according to PRPs.

Although PRPs agreed on many procedural aspects for future studies testing and evaluating the intervention, some issues remained undecided following this process. To further address parents’ preferences regarding study procedures, a cross-sectional, Web-based study has been conducted (results forthcoming) to examine their attitudes and preferences regarding, for example, the mode of study invitation, how study information should be presented and by whom, type of control conditions in a controlled study, and the acceptable waiting time in a controlled study with a wait-list control condition.

#### Process Evaluation

To facilitate the collaboration, continuous process evaluations were carried out. PRPs suggested some clarifications, for example, with regard to the time period of interest regarding their lived experience (eg, during treatment, at treatment completion, or at present) and according to which role they should provide feedback on the material (as hypothetical participants in a future study using the material as part of the intervention, or as research partners in the ongoing PAR process). PRPs were encouraged to approach the materials as research partners, but to give their opinion, they also needed to test the materials as hypothetical participants in a future study. Instructions were modified according to clarifications.

PRPs expressed concerns about working so closely with the facilitators who also created the materials for the workshops and Web-based exercises and whether this might influence their perspective as PRPs. To reduce potential respondent bias, the research group adapted the PAR process to include the presentation of materials by members of the research group not otherwise involved in the workshops. Furthermore, PRPs highlighted the psychological and emotional demands of taking part in the PAR process. However, having received CBT within our previous research [11,12] (personal communication by L Ljungman, 2017-10-23) was perceived as helpful in coping with these demands.

## Discussion

### Principal Findings

A 10-week internet-administered, CBT-based, psychological, guided, self-help intervention (ENGAGE) for parents of children previously treated for cancer, alongside procedures for future studies testing and evaluating the intervention, were developed in collaboration with PRPs and ERPs. Specifically, the content of the intervention, mode and frequency of e-therapist support, and the individualized approach for feedback were modified based on input from the PRPs or ERPs. Shared solutions were reached regarding the type and timing of support from an e-therapist, duration and timing of intervention, and the removal of “unnecessary” support functions. The ENGAGE intervention will include written, audio, and video materials as well as an initial support session via video or telephone in which individual problem analyses and idiographic goals will be formulated. The Web-based intervention will be delivered via the Portal with weekly e-therapist support. A “booster” session will be provided via video or telephone midway through the intervention. Participants will complete 1 module per week over a 10-week treatment period. Guidance from e-therapists will consist of 1 video- or telephone-assessment session, weekly Web-based written support, and a mid-treatment video- or telephone “booster” session. Module content will include, for example, psychoeducation, case vignettes, exercises based on specific CBT techniques, action plans, and questionnaires to assess symptoms. Preferences for study procedures in future studies testing and evaluating the intervention were discussed, but consensus was not reached. Overall, collaborative work added significantly to the study as a whole, with PRPs’ feedback continuously informing the research process, highlighting the value of working closely with the target population when conducting intervention research.

PRPs’ preferences regarding the delivery and content of the intervention are largely consistent with the existing, related literature for other study populations. For example, including patient vignettes in the intervention materials to facilitate normalization [24] and enhance identification with the intervention content [25]. Furthermore, PRPs’ concerns regarding limitations of not being able to personalize the content of the intervention are consistent with wider research [14]. Moreover, the lack of personal interaction with an e-therapist has been highlighted [26], with participants reporting difficulties developing a relationship with an e-therapist in the absence of face-to-face contact [27]. However, the inclusion of a video call at the beginning of the intervention may overcome these concerns. Indeed, the delivery of CBT via videoconference is as effective as face-to-face CBT [28], allowing similar communication to that in face-to-face therapy [29]. The inclusion of some form of face-to-face contact with an e-therapist may help develop a therapeutic alliance [30]. Furthermore, supported by the wider literature, was the suggestion to enhance the acceptance of the internet-administered intervention, and thus potentially facilitate recruitment, by presenting an informative video about internet-administered CBT to potential participants [31-33].

Although some findings were in line with existing related literature concerning the development of internet-administered interventions, this study further extends our understanding of acceptable intervention adaptation for the population. First, before the study, the research group posited that the inclusion of support functions (eg, a chat function, a forum, and a Q&A function) would be desirable and enhance intervention interactivity. However, PRPs felt such functions were unnecessary, and these were thus not included in the intervention. Second, PRPs were introduced to the conceptualization of distress experienced by parents of children previously treated for cancer, as informed by previous research conducted by our group [4,5,11,12] (personal communication by L Ljungman, 2017-10-23). Although PRPs reported that the conceptualization of distress was acceptable, and reflected their own experiences, a number of modifications were suggested concerning the adoption of “lay language,” as opposed to the psychological terminology used in the intervention material. As such, collaboration with PRPs further contributed to our understanding of how to communicate psychological principles to the population using more acceptable and accessible terminology.

The key elements of a PAR approach include understanding, mutual involvement, change, and a process that promotes personal growth [34]. Fulfillment of these elements could be identified from the PRPs’ responses to workshop evaluations. Establishing participants as equal research partners can be difficult, especially if participants experience low self-efficacy regarding their ability to participate in the process as equal partners [35]. Future PAR research may look to include training for research partners without research experience [36]. However, PRPs reported personal growth and helping others as significant motivators for contributing to the study.

### Strengths and Limitations

A strength of the study was the involvement of PRPs and ERPs via a PAR approach in developing the intervention ENGAGE and study procedures for future studies testing and evaluating the intervention. As such, the development was informed by previous research [4,5,11,12] (personal communication by L Ljungman, 2017-10-23), PRPs’ lived experience, and ERPs’ expert knowledge. We expect the importance of this approach to be reflected in the acceptability and feasibility of the intervention and study procedures in a forthcoming feasibility study. However, it should be noted that PRPs had received psychological support in previous intervention research. This means that they might have a more positive attitude toward research in comparison with the wider population and that the current findings may not describe the experiences of parents of children previously treated for cancer who have not accessed psychological support. It should also be considered that although PRPs of both genders and from different socioeconomic backgrounds were included, the sample was small and recruited from a small geographical area. In addition to inviting parents who had participated in our group’s previous intervention research, an open invitation to participate in the study was posted through the Swedish Childhood Cancer Foundation Website and the Swedish Childhood Cancer Association Middle-Sweden Facebook page. However, this recruitment strategy did not result in any further potential participants registering interest in participating in the study. As such, future research may benefit from adopting more assertive recruitment methods to identify researcher partners from the wider community of parents of children previously treated for cancer [36]. Although consensus was not always reached, in general, acceptable alternatives were agreed upon by PRPs. Ideas were discussed by PRPs and facilitators to generate shared solutions. In some cases, PRPs’ preferences were deemed unfeasible by the research group. For example, PRPs mentioned a preference for recruitment via hospital departments. However, as recruitment to the forthcoming feasibility study is planned for up to 5 years after end of a child’s treatment and parents are likely no longer in contact with the hospital department at that time, such a recruitment strategy is not possible to adopt.

### Conclusions

To the best of our knowledge, this study is the first use of a PAR approach to develop a CBT-based, internet-administered, guided, self-help intervention for parents of children previously treated for cancer and acceptable procedures for future studies testing and evaluating the intervention. We believe involvement of parents with lived experience and experts with expert knowledge have been vital in the development of a potentially relevant and acceptable intervention for this population. Specifically, the PAR process informed intervention content, including language, duration, mode, and frequency of e-therapist support. Furthermore, planned recruitment strategies for use in a planned feasibility study were modified, which may enhance participation, for example, via the inclusion of an informative video about internet-administered interventions. In addition, this planned feasibility study will further examine the relevance and acceptability of the developed intervention. The PAR process adopted in this study may inform the future use of PAR techniques to adapt and tailor internet-administered, psychological, guided, self-help interventions for other populations.

## Abbreviations

CBT: cognitive behavior therapy
ERP: expert research partner
PAR: participatory action research
PRP: parent research partner
PTSS: post-traumatic stress symptoms
Q&A: question and answer
RCT: randomized controlled trial
